# SARS-CoV-2 ORF7a Protein Impedes Type I Interferon-Activated JAK/STAT Signaling by Interacting with HNRNPA2B1

**DOI:** 10.3390/ijms26125536

**Published:** 2025-06-10

**Authors:** Yujie Wen, Chaochao Li, Tian Tang, Chao Luo, Shan Lu, Na Lyu, Yongxi Li, Rong Wang

**Affiliations:** Department of Laboratory Animal Science, School of Basic Medical Sciences, Xi’an Jiaotong University, Xi’an 710061, China; wenyujie1108@163.com (Y.W.); lcc120@stu.xjtu.edu.cn (C.L.); tangtian1118@163.com (T.T.); lc13312713816@163.com (C.L.); lushan15102@163.com (S.L.); lyuna2024@163.com (N.L.); lyx491838238@163.com (Y.L.)

**Keywords:** severe acute respiratory syndrome coronavirus 2, Janus kinase/signal transducer and activator of transcription (JAK/STAT) signaling, accessory protein ORF7a, heterogeneous nuclear ribonucleoprotein A2B1 (HNRNPA2B1)

## Abstract

The pandemic of Coronavirus Disease 2019 has triggered a worldwide public health emergency. Its pathogen, severe acute respiratory syndrome coronavirus 2 (SARS-CoV-2), has developed multiple strategies for effectively evading the host immune defenses, including inhibition of interferon (IFN) signaling. Several viral proteins of SARS-CoV-2 are believed to interfere with IFN signaling. In this study, we found that the SARS-CoV-2 accessory protein ORF7a considerably impaired IFN-activated Janus kinase/signal transducer and activator of transcription (JAK/STAT) signaling via suppression of the nuclear translocation of IFN-stimulated gene factor 3 (ISGF3) and the activation of STAT2. ORF7a dampened STAT2 activation without altering the expression and phosphorylation of Janus kinases (JAKs). A co-immunoprecipitation (co-IP) assay was performed to gather ORF7a protein, but it failed to precipitate STAT2. Interestingly, mass spectrometry and immunoblotting analyses of the ORF7a co-IP product revealed that ORF7a interacted with an RNA-binding protein, heterogeneous nuclear ribonucleoprotein A2B1 (HNRNPA2B1), and HNRNPA2B1 was related to the inhibitory effect of ORF7a on STAT2 phosphorylation. Moreover, examination of ORF7a deletion constructs revealed that the C-terminal region of ORF7a (amino acids 96 to 122) is crucial for suppressing IFN-induced JAK/STAT signaling activation. In conclusion, we discovered that SARS-CoV-2 ORF7a antagonizes type I IFN-activated JAK/STAT signaling by interacting with HNRNPA2B1, and the C-terminal region of ORF7a is responsible for its inhibitory effect.

## 1. Introduction

The epidemic of coronavirus disease 2019 (COVID-19), sparked by severe acute respiratory syndrome coronavirus 2 (SARS-CoV-2), has evoked an unseen and unparalleled threat to global health. The manifestations of COVID-19 range from asymptomatic or mild symptoms to severe pneumonia and even acute respiratory distress syndrome. SARS-CoV-2, a member of the *Betacoronaviruses* genus within the *Coronaviridae* family, is an enveloped positive-strand RNA virus. Its genome is approximately 30 kb in length and comprises 14 open reading frames (ORFs) to encode 29 viral proteins, including 4 structural proteins [spike (S), envelope (E), membrane (M), and nucleocapsid (N) proteins], 16 non-structural proteins (NSP1–16) and a series of accessory proteins (ORF3a, 3b, 6, 7a, 7b, 8, 9b, 9c, and 10) [1,2]. In addition to being involved in viral replication, transcription assembly and infection, several viral proteins also exert efforts to suppress the host immune response.

Interferons (IFNs), essential cytokines involved in host antiviral immune response, are primarily categorized into three types: I (IFNα and β), II (IFNγ), and III (IFNλ). Type I IFN plays a key role in establishing the host’s antiviral defense via initiating the Janus kinase/signal transducer and activator of transcription (JAK/STAT) signaling [3]. In the canonical type I IFN signaling pathway, IFNs bind to their receptors located on the cell surface, activating Janus kinases (JAKs), which then phosphorylate the downstream STAT1 and STAT2. Activated STAT1 and STAT2 form a heterodimer and recruit interferon-regulatory factor 9 (IRF9), leading to assembly of the STAT1/STAT2/IRF9 complex, known as IFN-stimulated gene factor 3 (ISGF3). ISGF3 then migrates into the nucleus and binds to interferon-stimulated response elements (ISREs), thereby initiating IFN-stimulated genes (ISGs) transcription. This process ultimately leads to the establishment of an antiviral state [3].

It is noteworthy that several viral proteins of SARS-CoV-2, especially the non-structural proteins (NSPs) and accessory proteins, are able to antagonize or hijack IFN-activated JAK/STAT signaling to facilitate viral replication and infection, contributing to immune escape [4,5,6]. SARS-CoV-2 accessory protein ORF7a plays an essential role in virus–host interactions, including regulation of autophagy, apoptosis, MHC-I-mediated antigen presentation, inflammatory response, and IFN signaling [7,8,9,10,11,12,13,14,15,16,17,18]. Cao et al. reported that SARS-CoV-2 usurped the host ubiquitin system to antagonize type I IFN signaling by ORF7a and that ubiquitination of ORF7a at Lys119 is critical for inhibiting STAT2 activation [19]. In this study, we found that SARS-CoV-2 ORF7a considerably impaired IFN-induced activation of JAK/STAT signaling by suppressing STAT2 phosphorylation and ISGF3 nuclear translocation. ORF7a had an interaction with heterogeneous nuclear ribonucleoprotein A2B1 (HNRNPA2B1), an RNA-binding protein, which is related to the inhibitory effect of ORF7a on STAT2 phosphorylation. The C-terminal region of ORF7a [amino acids (aa) 96–122] is indispensable for its inhibitory roles. This finding enhances our understanding of how SARS-CoV-2 evades the host antiviral immune response.

## 2. Results

### 2.1. ORF7a of SARS-CoV-2 Suppresses IFNα-Activated JAK/STAT Signaling

**Figure 1:**
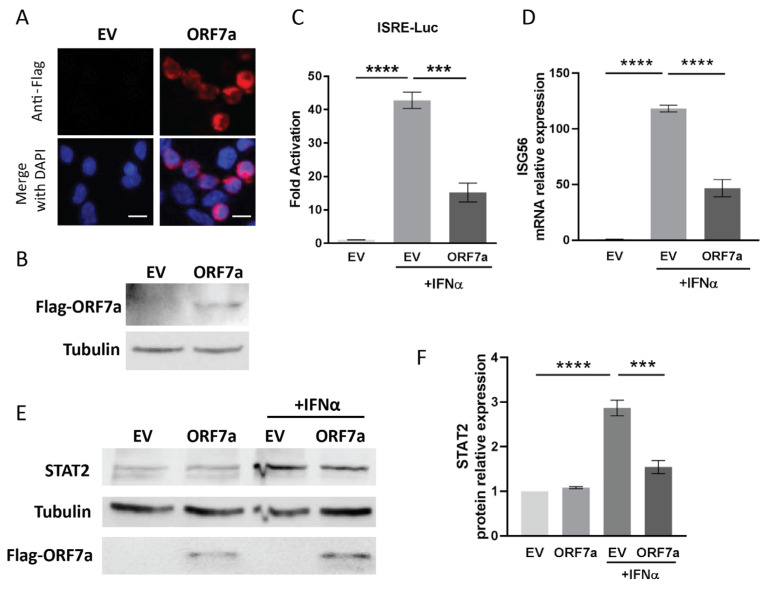
Accessory protein ORF7a of severe acute respiratory syndrome coronavirus 2 (SARS-CoV-2) suppresses interferon alpha (IFNα)-activated Janus kinase/signal transducer and activator of transcription (JAK/STAT) signaling. (**A**,**B**) Detection of ORF7a plasmid expression in HEK293T cells by immunofluorescence staining (**A**) and immunoblotting (**B**) with anti-Flag antibody. In (**A**), red fluorescence indicates ORF7a expression. Nuclear DNA labeled with 4′,6-diamidino-2-phenylindole (DAPI) (shown in blue). Scale bars = 10 µm. (**C**) Effect of ORF7a on IFN-stimulated response element (ISRE)-promoter activation. Cells were transfected with ISRE-Luc reporter and empty vector (EV) or ORF7a plasmid. Twenty-four hours later, IFNα was added into cell culture medium for additional 24 h stimulation. Luciferase activity was detected and the relative activation fold of ISRE-Luc reporter is shown. (**D**,**E**) Effect of ORF7a on the expressions of IFN-stimulated gene 56 (ISG56) and STAT2. Cells were transfected with EV or ORF7a plasmid for 24 h, followed by IFNα treatment for another 24 h. ISG56 mRNA levels (**D**) and STAT2 protein levels (**E**) were analyzed by RT-qPCR and immunoblotting, respectively. (**F**) Densitometry analysis showing the fold changes of STAT2 protein compared to the mock-treated EV control after normalization to tubulin. Error bars indicate the standard errors of the results obtained from three independent experiments. Statistical significance was determined using one-way ANOVA. *** *p* < 0.001, **** *p* < 0.0001.

It is reported that SARS-CoV-2 is able to antagonize JAK/STAT signaling by its accessory proteins [20,21]. In the present study, to assess the effect of SARS-CoV-2 accessory protein ORF7a on IFN signaling, ORF7a was expressed into HEK293T cells (Figure 1A,B) and an ISRE luciferase reporter assay was performed. Compared to the mock-treated cells with an empty vector (EV) transfection, IFNα stimulation led to 42.8-fold and 15.2-fold increase in ISRE reporter expression in EV- and ORF7a-plasmid-transfected cells, respectively (Figure 1C). However, the IFN-induced upregulation of ISRE reporter expression in ORF7a-transfected cells was obviously weaker than that in EV-transfected cells. This indicated that ORF7a significantly suppressed the IFN-induced ISRE reporter activation.

Once type I IFN signaling is activated, it will cause increased expressions of many IFN-responsive genes, including ISG56 and STAT2 [3]. Therefore, in order to ascertain the function of ORF7a on IFN signaling, RT-qPCR and immunoblotting were conducted to evaluate the expression levels of ISG56 and STAT2. In comparison with mock-treated EV control cells, exogenous IFNα stimulation resulted in a 118.2-fold and 46.8-fold increase in ISG56 transcript levels in EV- and ORF7a-transfected cells, respectively (Figure 1D). And the enhanced levels of ISG56 in cells with ORF7a transfection was considerably lower than that in IFNα-treated EV-transfected cells (Figure 1D). Similarly, in response to the IFNα treatment, the cells transfected with ORF7a plasmid exhibited significantly lower STAT2 level than EV-transfected cells (Figure 1E). Compared to mock-treated EV-transfected cells, STAT2 protein level was significantly upregulated to 2.9-fold in EV-transfected cells with IFNα treatment, while only a 1.5-fold elevation was observed in ORF7a-transfected IFNα-treated cells (Figure 1F). These results demonstrated that ORF7a played a remarkable inhibitory role on IFNα-activated JAK/STAT signaling.

### 2.2. ORF7a Suppresses IFNα-Induced JAK/STAT Signaling Activation via Blocking Nuclear Translocation of ISGF3

**Figure 2:**
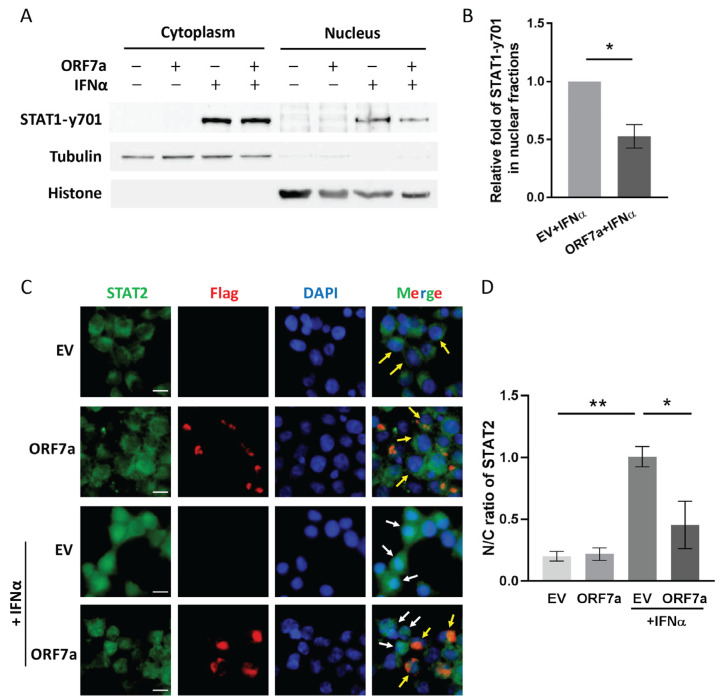
ORF7a suppresses IFNα-induced JAK/STAT signaling activation via restraining nuclear translocation of IFN-stimulated gene factor 3 (ISGF3) trimer. (**A**,**B**) ORF7a inhibits phosphorylated-STAT1 nuclear translocation. Cells were transfected with EV or ORF7a plasmid for 24 h, followed by stimulation with IFNα for 0.5 h. Then, cytoplasmic and nuclear fractions were isolated for immunoblotting with anti-STAT1-y701, anti-tubulin, and anti-histone antibodies (**A**). (**B**) Densitometry analysis showing the fold changes of STAT1-y701 in nuclear fractions after normalization to histone levels. Data are presented as means ± standard error of the mean (SEM) from three independent experiments. Statistical significance was determined by Student’s *t*-test. * *p* < 0.05. (**C**) ORF7a restrains the nuclear translocation of STAT2. After transfection with EV or ORF7a plasmid for 24 h, the cells were stimulated with IFNα for an additional 0.5 h and then fixed for immunofluorescence staining. Expressions of STAT2 and ORF7a (equipped with Flag tag) are shown in green and red fluorescence, respectively. Nuclear DNA was labeled with DAPI (blue). Arrows indicate the STAT2 localization in the cytoplasm (yellow) or nucleus (white). Scale bars = 10 µm. (**D**) Quantitative analysis of the content ratio of STAT2 in the cytoplasm and nucleus. Based on the fluorescence images shown in (**C**), the fluorescent intensities of STAT2 in the nucleus and cytoplasm were quantitated using ImageJ software, version 1.54g. One-way ANOVA was used for the statistical analysis. * *p* < 0.05, ** *p* < 0.01.

As type I IFNs attach to their receptors, the ISGF3 complex comprised of phosphorylated STAT1, phosphorylated STAT2, and IRF9 rapidly forms in the cytoplasm. ISGF3 complex translocation from cytoplasm to nucleus is crucial for the activation of IFN-induced JAK/STAT signaling. To explore whether ORF7a weakens IFN-activated signaling by inhibiting ISGF3 nuclear translocation, we conducted subcellular fractionations and immunoblotting to examine the intracellular distribution of phosphorylated STAT1 in IFN-stimulated cells with or without ORF7a transfection. In the absence of IFNα treatment, phosphorylated STAT1 at tyrosine 701 (STAT1-y701) was undetectable in the cells (Figure 2A). After IFNα stimulation, STAT1 was phosphorylated and partial STAT1-y701 translocated from cytoplasm to nucleus (Figure 2A). However, there was less STAT1-y701 existing in the nucleus of ORF7a-transfected cells than in the EV-transfected control (Figure 2A). Densitometry analysis of the nuclear fraction immunoblotting showed that STAT1-y701 level in the ORF7a transfected cells with IFNα treatment was significantly decreased to 0.5-fold compared to that in the IFNα-treated EV control cells (Figure 2B).

In addition, IFNα-induced STAT2 nuclear translocation was also examined by immunofluorescence assay. IFNα stimulation led to most STAT2 translocating into the nucleus in the EV-transfected cells, whereas most STAT2 was confined in the cytoplasm of the cells with ectopic ORF7a expression (Figure 2C,D). The above results indicated that ORF7a interfered with the IFN-induced activation of JAK/STAT signaling by dampening the nuclear migration of ISGF3.

### 2.3. ORF7a Impairs IFNα-Induced Phosphorylation of STAT2 Instead of STAT1

**Figure 3:**
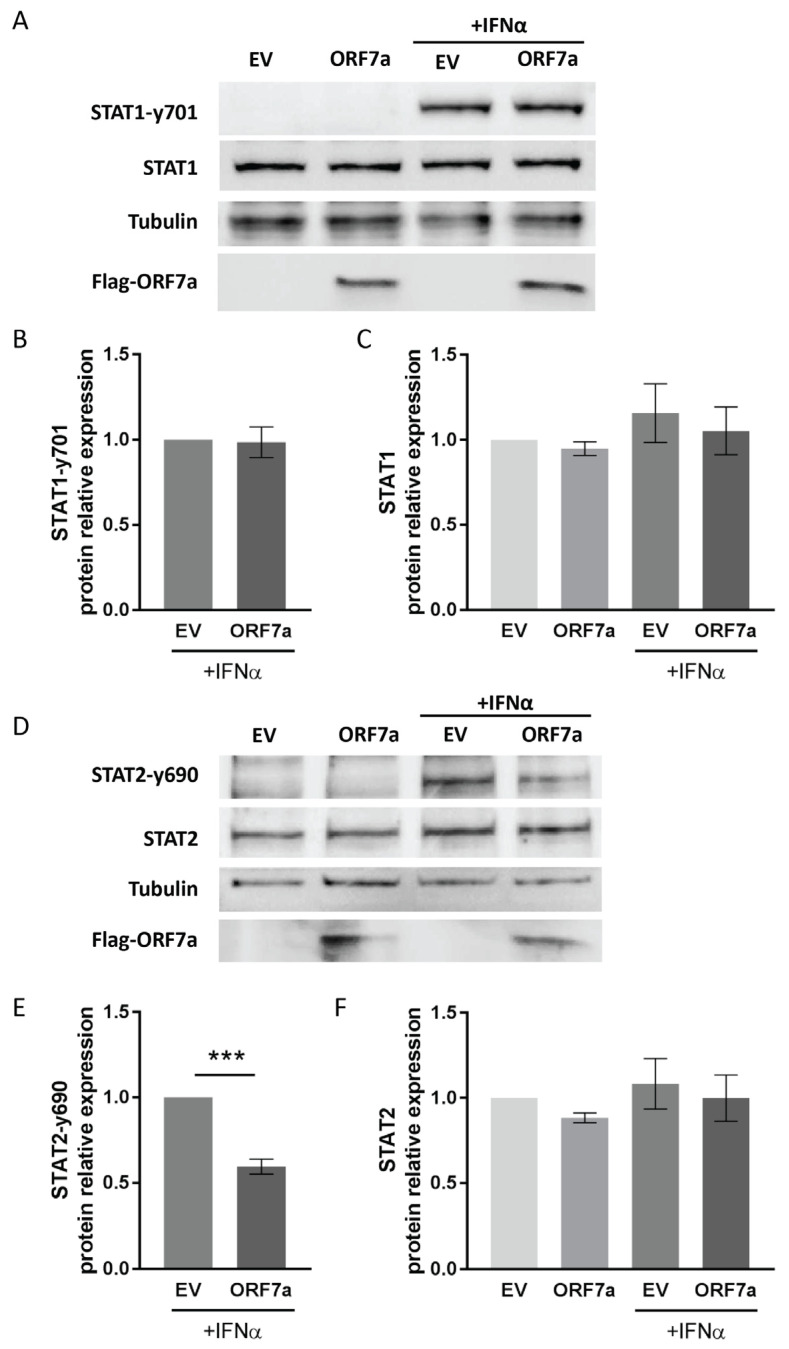
ORF7a impairs IFNα-induced phosphorylation of STAT2 instead of STAT1. (**A**) ORF7a has a marginal effect on the expression and activation of STAT1. Cells were transfected with EV or ORF7a plasmid. Twenty-four hours later, the cells were exposed to IFNα for 0.5 h and then harvested for immunoblotting with anti-STAT1-y701, anti-STAT1, anti-tubulin, and anti-Flag antibodies. (**B**,**C**) Densitometry analysis showing the relative fold changes of STAT1-y701 (**B**) and STAT1 (**C**) compared to the EV-transfected control cells after normalization to tubulin. (**D**) ORF7a suppresses STAT2 phosphorylation. The harvested whole cell lysates were also determined by immunoblotting with anti-STAT2-y690, anti-STAT2, anti-tubulin and anti-Flag antibodies. (**E**,**F**) Densitometry analysis showing the relative fold changes of STAT2-y690 (**E**) and STAT2 (**F**) compared to the EV-transfected control after normalization to the corresponding tubulin level. Data are presented as means ± SEM from three independent experiments. Statistical significance was determined by Student’s *t*-test (for comparisons of 2 groups) or one-way ANOVA (for comparisons of ≥3 groups). *** *p* < 0.001.

In response to IFNα stimulation, phosphorylation of STAT1 and STAT2 is a prerequisite for ISGF3 complex formation and its nuclear translocation. Therefore, we wondered whether ORF7a inhibited IFNα-activated signal transduction via interfering with STATs phosphorylation. The results showed that ORF7a had little impact on the expression and phosphorylation of STAT1 (Figure 3A–C), whereas it obviously decreased the phosphorylation levels of STAT2 (Figure 3D,E). As expected, the phosphorylated STAT2 at tyrosine 690 (STAT2-y690) was below the detectable level in mock-treated cells. In response to IFNα treatment, the STAT2-y690 level was notably elevated in cells with EV transfection, while STAT2 phosphorylation levels were significantly reduced to 0.6-fold compared to IFNα-treated EV control cells (Figure 3E). But ORF7a had a minimal impact on the total protein levels of STAT2 (Figure 3F). The result above demonstrates that ORF7a suppresses the phosphorylation of STAT2 rather than STAT1, which is linked to the inhibitory effect of ORF7a on IFN-activated signaling.

### 2.4. HNRNPA2B1 Interacts with ORF7a and Is Related to the Inhibitory Effect of ORF7a on STAT2 Phosphorylation

**Figure 4:**
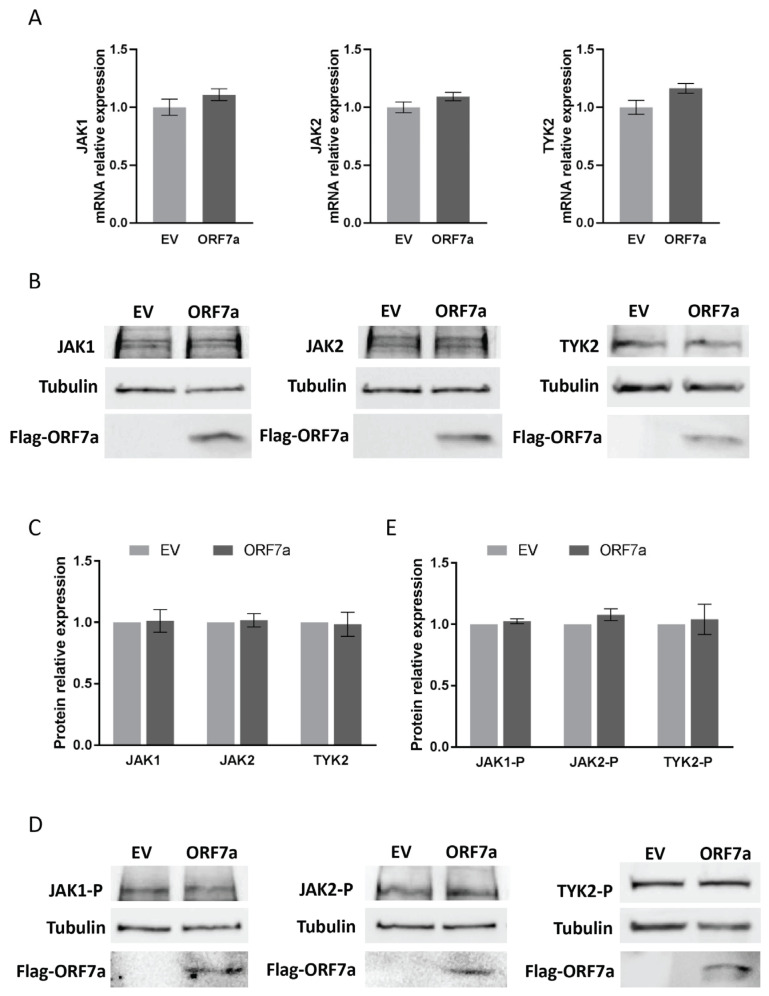
ORF7a has no effect on Janus kinases (JAKs). Cells were transfected with EV or ORF7a plasmid for 24 h, then stimulated with IFNα. After 24 h IFN-stimulation, the mRNA (**A**) and protein (**B**) levels of JAK1, JAK2, and TYK2 were evaluated. (**C**) Densitometry analysis showing the relative fold changes of JAK1, JAK2, and TYK2 total protein levels. (**D**) The phosphorylation levels of JAK1 (JAK1-P), JAK2 (JAK2-P), and TYK2 (TYK2-P) after 0.5 h of IFN-stimulation. (**E**) Densitometry analysis showing the relative fold changes of JAK1-P, JAK2-P, and TYK2-P. Relative fold changes are shown after normalization to the corresponding tubulin level. Error bars indicate the standard errors of the results obtained from three independent experiments. Student’s *t*-test was used for statistical analyses.

JAKs (specifically JAK1, JAK2, and TYK2) play a crucial role in the phosphorylation of STATs [3]. To identify whether ORF7a suppressed STAT2 phosphorylation via interfering with JAKs, we assessed the expression and activation of JAKs in ORF7a-transfected cells using RT-qPCR and immunoblotting. In comparison with EV-transfected cells, the transcript (Figure 4A) and protein (Figure 4B,C) levels of JAK1, JAK2, and TYK2 showed minimal changes in the ORF7a-transfected cells. Since the phosphorylation level of JAKs reflects their own activation level, immunoblotting was also used to examine the phosphorylation levels of JAK1 (JAK1-P), JAK2 (JAK2-P), and TYK2 (TYK2-P). Compared to EV control cells, the levels of JAK1-P, JAK2-P, and TYK2-P were similar to those in cells with ORF7a expression (Figure 4D,E). These results indicate that ORF7a has no effect on JAKs expression and activation.

**Figure 5:**
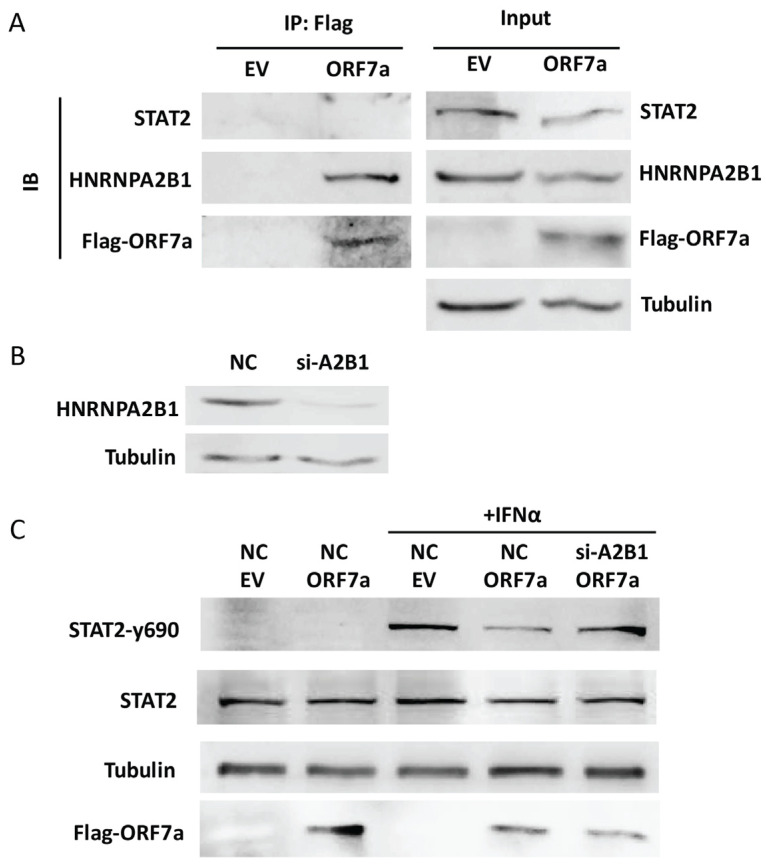
Heterogeneous nuclear ribonucleoprotein A2B1 (HNRNPA2B1) interacts with ORF7a and is related to the inhibition of STAT2 phosphorylation by ORF7a. (**A**) ORF7a interacts with HNRNPA2B1 rather than STAT2. Cells were transfected with either EV or ORF7a plasmid (equipped with a Flag tag). After 24 h, the cells were stimulated with IFNα for an additional 0.5 h and then harvested for co-immunoprecipitation (co-IP) with anti-Flag IP resin. The input and IP products were analyzed by immunoblotting (IB) with anti-STAT2, anti-HNRNPA2B1, anti-Flag, and anti-tubulin antibodies. (**B**) The knockdown effect of si-HNRNPA2B1 on HNRNPA2B1 expression. Cells were transfected with non-targeting control siRNA (NC) or si-HNRNPA2B1 (si-A2B1) (100 nM). Twenty-four hours later, the cells were harvested for immunoblotting with anti-HNRNPA2B1 and anti-tubulin antibodies. (**C**) Silencing of HNRNPA2B1 abolishes the inhibitory effect of ORF7a on STAT2 phosphorylation. Cells were transfected with EV or ORF7a plasmid along with NC or si-A2B1. At 24 h post-transfection, the cells were stimulated with IFNα (0.5 h) and then harvested for immunoblotting with antibodies against STAT2-y690, STAT2, tubulin, and Flag. Representative images from three independent experiments are shown.

To identify whether ORF7a suppressed IFNα-induced STAT2 phosphorylation via an interaction with STAT2, co-IP and immunoblotting were conducted. We transfected HEK293T cells with either the EV or ORF7a plasmid (equipped with a Flag tag). 24 h later, the cells were stimulated with IFNα for half an hour and then subjected to co-IP with anti-Flag IP resin to gather ORF7a protein. The co-IP products were analyzed using immunoblotting with anti-STAT2 antibody. The result showed that there was no STAT2 band visible (Figure 5A). This suggests that there is no interaction between ORF7a and STAT2. To identify which proteins mediate the ORF7a-induced decrease in STAT2 phosphorylation, the co-IP products harvested from EV and ORF7a-transfected cells were subjected to a mass spectrometry analysis. The result showed that compared to the EV-transfected cells, nine proteins with at least five unique peptides were exclusively present in the samples harvested from the ORF7a-transfected cells (Table 1). Among the nine proteins, HNRNPA2B1, an RNA-binding protein, is known to play a vital role in the antiviral innate immune response [22,23]. Zhou et al. reported that knockdown of HNRNPA2B1 significantly enhanced STAT2 activation upon IFNα stimulation [24]. Therefore, we speculated that ORF7a reduced STAT2 phosphorylation via interacting with HNRNPA2B1. To verify the interaction between ORF7a and HNRNPA2B1, we performed co-IP using anti-Flag IP resin on EV or ORF7a-transfected cells after 0.5 h IFNα-treatment, and the co-IP products were analyzed using immunoblotting with anti-HNRNPA2B1 antibody. We found that there was indeed an interaction between ORF7a and HNRNPA2B1 (Figure 5A). To further investigate whether HNRNPA2B1 is related to the inhibitory effect of ORF7a on STAT2 phosphorylation, we knocked down the expression of HNRNPA2B1 using siRNA (Figure 5B) and then assessed the phosphorylation level of STAT2. This showed that siRNA silencing of HNRNPA2B1 abolished the inhibitory effect of ORF7a on STAT2 activation. In response to the IFNα stimulation, compared to the non-targeting control siRNA (NC)-transfected cells with ORF7a expression, the cells co-transfected with ORF7a and si-HNRNPA2B1 (si-A2B1) showed a considerably higher level of STAT2-y690, which was similar to that in the cells co-transfected with EV and NC (Figure 5C). These results demonstrate that ORF7a might inhibit STAT2 activation via interacting with HNRNPA2B1.

### 2.5. The C-Terminal Domain of ORF7a (Aa 96–122) Appears to Be Responsible for the IFN Signaling Inhibition

**Figure 6:**
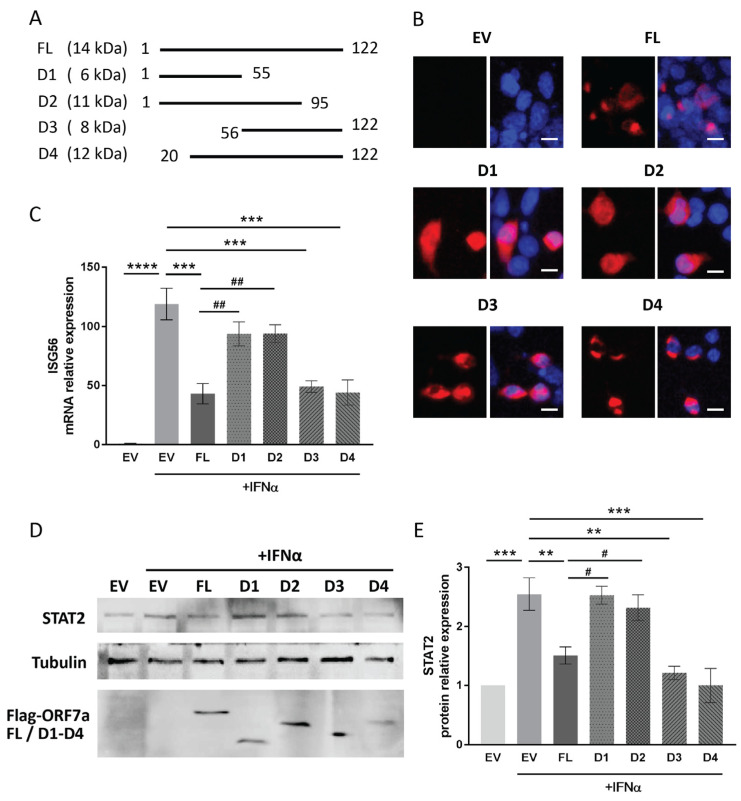
The C-terminal region of ORF7a appears to be responsible for the IFN signaling inhibition. (**A**) Schematic illustration of ORF7a truncation constructs. FL indicates full length of ORF7a. The numbers by the lines mark the start and end amino acid positions of ORF7a in each construct (D1–D4). (**B**) Detection of the ORF7a deletion constructs expression in HEK293T cells using immunofluorescence staining with anti-Flag antibody. Expressions of ORF7a FL and deletion constructs are shown in red fluorescence. Nuclear DNA labeled with DAPI (blue). Scale bars = 10 µm. (**C**,**D**) The impact of ORF7a truncation constructs on IFN signaling activation. Cells were transfected with ORF7a FL or truncation constructs. Twenty-four hours later, IFNα was added into cell culture for additional 24 h stimulation, followed by detection of ISG56 transcription levels (**C**) and STAT2 protein levels (**D**), respectively. (**E**) Densitometry analysis showing the relative fold changes in STAT2 levels compared to the levels in the mock-treated EV control cells after normalization to tubulin. Data are presented as means ± SEM from three independent experiments. One-way ANOVA was used for statistical analyses. ** *p* < 0.01, *** *p* < 0.001, **** *p* < 0.0001 vs. EV + IFNα group; # *p* < 0.05, ## *p* < 0.01 vs. FL + IFNα group.

For screening the functional domain of ORF7a involved in the suppression of IFN signaling, four deletion constructs of ORF7a (D1–D4) were prepared (Figure 6A). Since the deletion constructs were equipped with Flag tag, their expression in HEK293T cells was verified using immunofluorescence staining with anti-Flag antibody (Figure 6B).

It is known that prolonged stimulation by IFN can lead to continuous activation of the IFN signaling pathway, resulting in increased transcription of downstream effector genes. Therefore, after 24 h of IFNα stimulation, we measured the IFNα-induced elevation levels of ISG56 and STAT2 in cells transfected with ORF7a truncation constructs. Compared to mock-treated cells, IFNα stimulation resulted in a 43.1-, 49.1-, and 44.1-fold increase in ISG56 mRNA levels in cells transfected with full-length ORF7a (FL), D3 (aa 56–122), and D4 (aa 20–122), respectively. These increases were significantly lower than the 118.9-fold elevation observed in IFNα-treated EV-transfected cells (Figure 6C). In contrast, ORF7a D1 (aa 1–55) and D2 (aa 1–95) had much less effect on IFN-induced ISG56 elevation (Figure 6C). Similarly, in response to 24-h stimulation of IFNα, the cells with ORF7a D3 or D4 transfection did not exhibit increased levels of STAT2 expression as in the IFNα-treated EV control (Figure 6D). The IFNα stimulation caused an approximate 2.5-fold upregulation of STAT2 level in EV-transfected cells, whereas ORF7a D3 and D4 constructs attenuated the IFNα-induced elevation in STAT2 expression (Figure 6E). The results above indicate that the C-terminal region (aa 96–122) of ORF7a had a suppressive impact on IFN-activated signaling.

**Figure 7:**
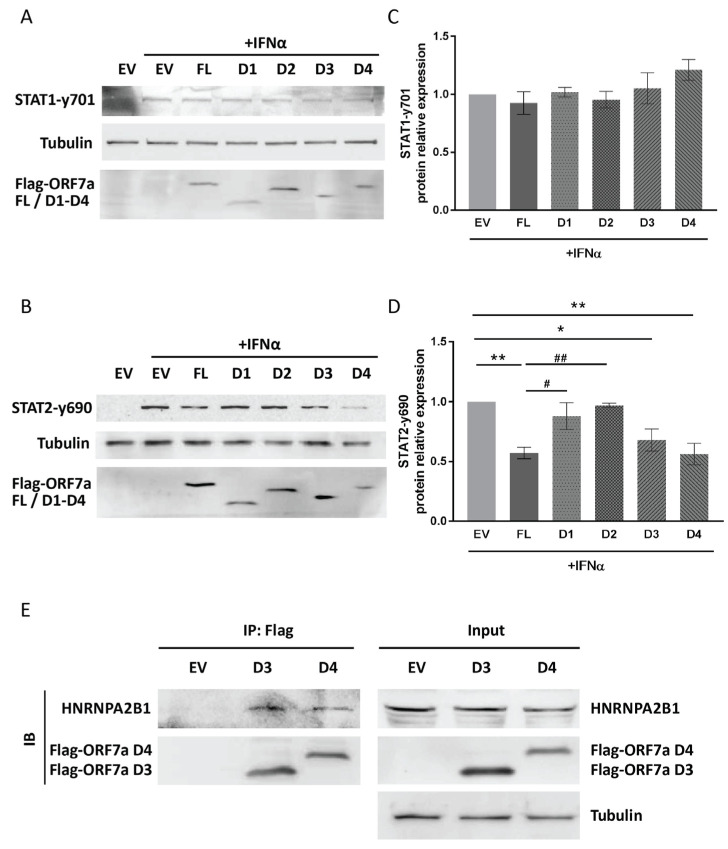
The C-terminal domain of ORF7a is related to the restraint of STAT2 phosphorylation. Cells were transfected with EV, ORF7a FL, or ORF7a truncation constructs. Twenty-four hours later, the cells were exposed to IFNα (0.5 h) and then harvested to determine the levels of STAT1-y701 (**A**) and STAT2-y690 (**B**). (**C**,**D**) Densitometry analysis showing the relative fold changes in STAT1-y701 (**C**) and STAT2-y690 (**D**) compared to the levels in IFNα-stimulated EV control cells after normalization to tubulin. Data are presented as means ± SEM from three independent experiments. Statistical significance was determined by one-way ANOVA. * *p* < 0.05, ** *p* < 0.01 vs. EV + IFNα group; # *p* < 0.05, ## *p* < 0.01 vs. FL + IFNα group. (**E**) Determining the interaction between HNRNPA2B1 and ORF7a D3 or D4. Cells were transfected with EV, ORF7a D3, or D4 plasmid. After 24 h, the cells were stimulated with IFNα for an additional 0.5 h and then harvested for co-IP with anti-Flag IP resin. The input and IP products were analyzed by immunoblotting (IB) with anti-HNRNPA2B1, anti-Flag, and anti-tubulin antibodies. Representative images from three independent experiments are shown.

To evaluate the effect of ORF7a deletion constructs on STAT1 and STAT2 activation, the phosphorylation levels of these proteins were measured. Immunoblotting results showed that all the truncated ORF7a constructs had no significant effect on IFNα-induced STAT1 phosphorylation (Figure 7A,C). However, in response to IFNα treatment, the cells with ORF7a D3 or D4 expression exhibited significantly lower levels of STAT2-y690 than the EV-transfected control cells (Figure 7B). The STAT2-y690 level in the IFNα-treated cells with ORF7a D3 or D4 expression were 0.7- and 0.6-fold of the levels observed in the IFNα-treated EV control cells, respectively (Figure 7D). Furthermore, co-IP was performed to determine the interaction between HNRNPA2B1 and ORF7a D3 or D4. After transfection with D3 or D4 plasmid for 24 h, the cells were stimulated by IFNα for an additional 0.5 h. Anti-Flag IP resin was used to gather D3 and D4 proteins. Then, the co-IP products were analyzed using immunoblotting with anti-HNRNPA2B1 antibody. We found that ORF7a D3 and D4 interacted with HNRNPA2B1 (Figure 7E). The results above suggested that the C-terminal region (aa 96–122) of ORF7a was involved in its interaction with HNRNPA2B1 and participated in the inhibition of STAT2 phosphorylation, thereby impeding JAK/STAT signaling activation.

## 3. Discussion

The interferon response constitutes the first line of the host antiviral defense. SARS-CoV-2 has evolved several strategies to effectively evade type I IFN-activated host innate immunity, including blocking IFN production, subverting IFN-activated downstream signaling, and antagonizing the antiviral activities of IFN-inducible genes. In addition to counteracting host antiviral defenses by dysregulating autophagy [15], apoptosis [16], and MHC-I-mediated antigen presentation [14], ORF7a concurrently disrupts type I interferon signaling to facilitate immune evasion [11,19]. It is reported that ORF7a hijacks the host ubiquitin system to poly-ubiquitinate itself, thereby suppressing IFN-induced STAT2 activation [19]. ORF7a also interacts with tetherin, which is encoded by an IFN-inducible gene [12]. ORF7a disrupts tetherin glycosylation, thereby partially counteracting the antiviral activity of tetherin [12,13]. In the current investigation, we discovered that SARS-CoV-2 ORF7a interacts with HNRNPA2B1, which contribute to the reduction of STAT2 phosphorylation, sequentially suppressing IFN signaling activation. Moreover, the C-terminal region (aa 96–122) of ORF7a plays a crucial role in inhibiting IFN-activated signaling.

For facilitating viral infection and replication, several SARS-CoV-2 proteins have been shown to antagonize IFN-activated signaling, such as NSPs (NSP1, 6, 13, 14), structural proteins (M, N), and accessory proteins (ORF3a, 6–8) [8,9,11,20,21,25,26,27,28]. They exhibited a capacity to repress the activation of the ISRE-promoter triggered by type I IFN. Although a previous study showed that SARS-CoV-2 ORF7a notably inhibited ISRE-promoter activation [11], there are still some other reports showing that ORF7a had a marginal effect on ISRE reporter [8,9,10]. Thus, in the present study, to assess the effect of ORF7a on type I IFN-activated signaling, not only was the ISRE reporter activation examined by performing luciferase reporter assay but the expressions of IFN-responsive genes were also evaluated using RT-qPCR and immunoblotting. We noticed that ORF7a significantly and consistently suppressed the IFN-activated signaling across all three detection methods.

Several viral proteins of SARS-CoV-2 antagonize IFN signaling by directly targeting key players of the IFN signaling cascade. For example, NSP14 diminishes the IFN’s effect by inducing IFN receptor degradation and abolishing ISGs induction [25,26]. ORF3a promotes JAK2 ubiquitination and subsequent degradation by upregulating the suppressor of cytokine signaling 1 (SOCS1), thereby interfering with the type I IFN signaling activation [21]. NSP1 induces STAT2 depletion to diminish ISG induction [8]. Structural protein M impairs STAT1 activation via directly interacting with STAT1 and prompting STAT1 autophagic degradation [28]. NSP6, NSP13, and N suppress STATs phosphorylation, thus impairing JAK/STAT activation [11,27]. ORF6 hijacks the trafficking of ISGF3 by directly binding to KPNA2 (a nuclear import) and Nup98-Rae1 (a component of nuclear pore complex) [9,11,20]. In this study, we noticed that ORF7a restrained nuclear translocation of ISGF3, and ORF7a selectively inhibited IFNα-induced phosphorylation of STAT2, but not STAT1. These findings were consistent with previous observations [11,19].

In addition, we discovered that ORF7a had no effect on the expression and activation of JAKs, but it interacted with HNRNPA2B1, an RNA-binding protein. It is known that HNRNPA2B1 not only regulates the transcription, splicing, transport, stability, and translation of a variety of RNAs but also plays a role in antiviral immune response [22,23]. For example, HNRNPA2B1 regulates nuclear-cytoplasmic transport processes to influence the polyribosomal distribution of HIV-1 RNA [29]. In response to DNA virus infection, HNRNPA2B1 acts as a DNA sensor that activates and amplifies type I IFN responses [30]. Zuo et al. reported that an HNRNPA2B1 agonist effectively reduced viral loads and attenuated lung inflammation in a SARS-CoV-2-infected hamster [31], suggesting that HNRNPA2B1 is related to SARS-CoV-2 replication and pathogenesis. Zhou et al. [24] found that SARS-CoV-2 NSP1 directly bonds with HNRNPA2B1 and the knockdown of HNRNPA2B1 significantly boosted STAT2 phosphorylation upon IFNα stimulation. This implies that HNRNPA2B1 is implicated in NSP1-induced disruption of type I IFN signaling. Here, by performing mass spectrometry and co-IP analyses, we screened out HNRNPA2B1 existing in the ORF7a co-IP product and confirmed HNRNPA2B1 binding to ORF7a. Moreover, silencing of HNRNPA2B1 with siRNA abolished the inhibitory effect of ORF7a on STAT2 phosphorylation. This suggests that ORF7a may suppress IFNα-induced STAT2 phosphorylation via interacting with HNRNPA2B1, which provides a potential explanation for the inhibitory effect of ORF7a on IFN signaling.

SARS-CoV-2 ORF7a truncations have emerged frequently worldwide. Many ORF7a deletion variants have been identified from clinical specimens, and the deletion mutations mainly occur at the C-terminal end of ORF7a [32,33,34,35,36,37,38,39]. An in silico analysis predicted that deletions in ORF7a could impact its structure and further impair its function [40]. It has been reported that the deletion of ORF7a reduces virus infectivity in cultured cells [40], and even small deletions (six or seven nucleotides deletion) within the C-terminal region are capable of delaying SARS-CoV-2 replication [41]. This suggests that mutations occurring in ORF7a may limit viral infection and replication. Furthermore, Nemudryi et al. pointed out that SARS-CoV-2 isolates with C-terminal deletions in ORF7a triggered an elevated type I interferon response to the viral infection [35], implying that truncations in the ORF7a C-terminus are related to viral immune modulation. Consistently, in this study, by utilizing four deletion constructs of ORF7a (D1–D4), we found that the C-terminal domain of ORF7a (aa 96–122) is critical for its inhibitory effect on IFN signaling. Similar to full-length ORF7a, truncations containing aa 96–122 significantly suppressed IFNα-induced elevation in ISG56 and STAT2 expression, as well as STAT2 phosphorylation. This indicates that the ORF7a C-terminal domain is responsible for ORF7a-induced immune suppression, which may explain why C-terminal-truncated ORF7a variants quickly disappear in the immunocompetent population [35].

## 4. Materials and Methods

### 4.1. Cells, Plasmids, siRNA and Reagents

HEK293T cells (sourced from ATCC, Manassas, VA, USA) were maintained in Dulbecco’s Modified Eagle’s Medium containing 10% fetal bovine serum (Cat# SV30087, HyClone, Logan, UT, USA) in a humidified atmosphere of 5% carbon dioxide at 37 °C.

Based on the sequence of SARS-CoV-2 Wuhan-Hu-1 strain (GenBank# NC_045512.2), the SARS-CoV-2 ORF7a sequence was synthesized by GenScript (Nanjing, China) and subsequently subcloned into pCAGEN-Flag vector as previously described [21,42]. The plasmid expressing the SARS-CoV-2 ORF7a protein was named pCAGEN-Flag-ORF7a. Deletion fragments of ORF7a were amplified from pCAGEN-Flag-ORF7a using the primers listed in Table S1, then cloned into the pCAGEN-Flag vector and named D1 to D4, respectively. Primers ORF7a-F and ORF7a-55R were used to amplify fragment D1; ORF7a-F and ORF7a-95R for D2; ORF7a-56F and ORF7a-R for D3; ORF7a-20F and ORF7a-R for D4. Restriction enzyme digestion and DNA sequencing were used to validate the correctness of the obtained recombinant plasmids.

The sequences of siRNA targeting human HNRNPA2B1 (si-HNRNPA2B1) and non-targeting control siRNA (NC) were 5′-CUUUGGUGGUAGCAGGAACTT-3′ [30] and 5′-UUCUCCGAACGUGUCACGUTT-3′, respectively. The siRNA duplexes were synthesized and purified by Sangon Biotech (Shanghai, China).

Lipofectamine™ 2000 transfection reagent (Cat# 11668-027, Invitrogen, Carlsbad, CA, USA) was used to transfect plasmids into cells according to the manufacturers’ instructions. Briefly, for each well, 1 μg of plasmid DNA and 3 μL of transfection reagent were diluted in Opti-MEM medium (Cat# 31985-070, Gibco, Grand Island, NY, USA). Diluted DNA and diluted transfection reagent were gently mixed and incubated for 5 min at room temperature. Then, we added the DNA-Lipofectamine complexes to each well. HiPerFect transfection reagent (Cat# 301705, QIAGEN, Shanghai, China) was used to transfect siRNA into cells. SiRNA (100 nM per well) and 6 μL of transfection reagent were mixed in Opti-MEM medium. After 5 min of incubation at room temperature, the mixture was added into each well. Recombinant human IFNα-2a was purchased from GenScript (Cat# Z03003-50, Nanjing, China) and used for cell stimulation (final concentration: 500 U/mL).

### 4.2. Immunofluorescence Assay

Immunofluorescence staining was conducted following the previous description [21,42]. Briefly, cells were fixed with 4% paraformaldehyde for 15 min, followed by permeabilization with 0.5% Triton X-100 for 10 min. Then, 10% normal goat serum was used to block cells for 0.5 h, followed by incubation with primary antibodies against STAT2 (Cat# ab32367, Abcam, Cambridge, UK, 1:50) or Flag (Cat# 66008, Proteintech, Chicago, IL, USA, 1:500) overnight at 4 °C. Secondary antibodies conjugated with Alexa Fluor 488 or Cy3 [Cat# 4412S, Cell Signaling Technology (CST), Danver, MA, USA, 1:300; Cat# ab97035, Abcam, Cambridge, UK, 1:200] were used for detection of the specific reactions. Nuclear DNA was stained with 4′,6-diamidino-2-phenylindole (DAPI) (Cat# C1002, Beyotime Biotech, Shanghai, China). A fluorescence microscope equipped with a digital camera (Olympus CKX53, Tokyo, Japan) was used for observation and photography.

### 4.3. Luciferase Reporter Assay

The experiment was performed as previously described [21,43]. Briefly, ISRE-promoter luciferase reporter plasmid (ISRE-Luc) and the test plasmids were transfected into cells. A Renilla luciferase vector was also co-transfected for normalization. After 24 h, the cells were stimulated with IFNα for additional 24 h and then examined using a Dual-Glo luciferase assay kit purchased from Promega (Cat# E2920, Madison, WI, USA). Compared to the mock-treated control, relative folds of ISRE reporter activity were shown after normalization against Renilla reporter values.

### 4.4. RNA Isolation, Reverse Transcription and Quantitative PCR (RT-qPCR)

RNA extraction and reverse transcription were conducted using RNAsio plus reagent (Cat# 9108, Takara, Kusatsu, Japan) and RT reagent Kit (Cat# RR047A, Takara, Kusatsu, Japan), respectively. A 2x SYBR GREEN mix (Cat# RK21203, Abclonal, Wuhan, China) was used in quantitative PCR. The primer sequences are shown in Table S1. Ribosomal protein L32 (*Rpl32*), a house-keeping gene, was also included for normalization of the total input RNA. The 2^−ΔΔCt^ threshold cycle method [44] was used to quantify the transcript levels, and relative fold changes compared to the control group were presented.

### 4.5. Immunoblotting

Protein samples were separated in 12% polyacrylamide gel, then transferred onto PVDF membranes for immunoblotting analysis as described previously [21,42]. After blocking with 5% skim milk in TBS-T (0.05% Tween 20 in Tris buffered saline), the membranes were incubated with primary antibody overnight at 4 °C. The following primary antibodies were used: STAT1 (Cat# 14994T, CST, Danver, MA, USA, 1:1000), phospho-STAT1 (STAT1-y701) (Cat# ab109457, Abcam, Cambridge, UK, 1:1000), STAT2 (Cat# ab32367, Abcam, Cambridge, UK, 1:5000), phospho-STAT2 (STAT2-y690) (Cat# ab191601, Abcam, Cambridge, UK, 1:1000), β-tubulin (Cat# HC101, TransGen Biotech, Beijing, China, 1:1000), Histone H2A (Cat# 7631, CST, Danver, MA, USA, 1:1000), JAK1 (Cat# ab133666, Abcam, Cambridge, UK, 1:1000), phosphorylated JAK1 (JAK1-P) (Cat# ab138005, Abcam, Cambridge, UK, 1:1000), JAK2 (Cat# ab108596, Abcam, Cambridge, UK, 1:5000), phosphorylated JAK2 (JAK2-P) (Cat# WL02997, Wanlei bio, Shenyang, China, 1:500), TYK2 (Cat# A2128, Abclonal, Wuhan, China, 1:500), phosphorylated TYK2 (TYK2-P) (Cat# bs-3437R, Bioss Inc., Woburn, MA, USA, 1:1000), HNRNPA2B1 (Cat# AY2564, Abways, Beijing, China, 1:500), and Flag (Cat# 66008, Proteintech, Chicago, IL, USA, 1:5000). The antibodies bound on the membrane were detected using secondary antibodies conjugated with horseradish peroxidase (Cat# HS101, HS201, TransGen Biotech, Beijing, China, 1:5000) and were revealed using a chemiluminescence substrate. The chemiluminescent signal was digitally recorded using the Chemi-Doc XRS imaging system (Bio-Rad, Hercules, CA, USA). Quantity One software, version 4.6.6 (Bio-Rad, Hercules, CA, USA) was used for digital image acquisition and analysis.

### 4.6. Subcellular Fractionation

A nuclear and cytoplasmic protein extraction kit (Cat# P0027, Beyotime Biotech, Shanghai, China) was utilized to extract subcellular fractions from cells. The harvested cytoplasmic and nuclear proteins were detected using immunoblotting. Anti-β-tubulin (Cat# HC101, TransGen Biotech, Beijing, China, 1:1000) and anti-histone H2A (Cat# 7631, CST, Danver, MA, USA, 1:1000) antibodies were used to assess the fractionation.

### 4.7. Co-Immunoprecipitation (Co-IP)

The assay was carried out following the previous description with some modifications [42]. A lysis buffer [42] containing protease and phosphatase inhibitor (Cat# 4693132001, 4906837001, Roche, Basel, Switzerland) was used to lyse cells. The lysate was centrifuged and the supernatant was incubated with an anti-Flag IP Resin (Cat# L00425, GenScript, Nanjing, China). Laemmli buffer (Cat#1610737, Bio-Rad, Hercules, CA, USA) was used to elute proteins from resin pellets. The eluted protein samples were analyzed by immunoblotting using anti-STAT2, anti-HNRNPA2B1 (Cat# AY2564, Abways, Beijing, China, 1:1000), and anti-Flag antibodies.

### 4.8. Statistical Analysis

Data were analyzed using GraphPad Prism 7.0 software and expressed as mean ± standard error of the mean (SEM). For comparison between 2 groups, Student’s *t*-test was used for statistical analyses, while for comparisons among 3 or more groups, one-way ANOVA was applied. *p* < 0.05 was considered statistically significant.

## 5. Conclusions

IFN response is indispensable for a host to establish antiviral defenses. To evade the immune response, viruses target IFN signaling pathways. Several viral proteins of SARS-CoV-2 are known to interfere with IFN-activated signaling. In this study, we discovered that SARS-CoV-2 ORF7a obviously impeded IFN-activated signaling by suppressing STAT2 phosphorylation and ISGF3 nuclear translocation. ORF7a bound to HNRNPA2B1, which contributed to the reduced STAT2 phosphorylation. Moreover, the C-terminal region (aa 96–122) of ORF7a is responsible for its inhibitory effect. The finding contributes to our understanding of SARS-CoV-2 evading host antiviral immunity.

## Figures and Tables

**Table 1:** Brief results of mass spectrometry.

Protein Name	Protein Score	Coverage (%)	Peptides	Unique Peptides
MYH10	785	12	21	17
HSP90AB1	574	26	16	7
HSPA8	427	17	8	6
MYL6	423	40	6	6
HNRNPA2B1	342	22	6	6
VCP	328	10	7	7
RPS3	254	21	5	5
CANX	197	11	7	7
HSP90B1	197	8	6	5

Note: Cells were transfected with pCAGEN-Flag-ORF7a or empty vector (EV). Twenty-four hours later, the cells were exposed to IFNα (0.5 h) and then harvested for IP with anti-Flag IP resin. The IP products were subjected to polyacrylamide gel electrophoresis, and then the gels were digested for mass spectrometry (MS). Proteome Discoverer 1.3 was used to process the MS results and Tandem mass spectra were searched against human SwissPort database (Homo_sapiens_9606_SP_20230103). Compared to the EV group, proteins with ≥5 unique peptides in the ORF7a-transfected cells were listed in the table, arranged in descending order of the protein score. The score indicates protein matching degree (the higher score, the better match).

## Data Availability

All data generated or analyzed during this study are included in this published article.

## Supplementary Materials

The following supporting information can be downloaded at: https://www.mdpi.com/article/10.3390/ijms26125536/s1.
