# Supplementary material for: SARS-CoV-2 ORF7a Protein Impedes Type I Interferon-Activated JAK/STAT Signaling by Interacting with HNRNPA2B1
Source: Int J Mol Sci. 2025 Jun 10;26(12):5536. doi: 10.3390/ijms26125536 (PMC12193315; doi:10.3390/ijms26125536)
Supplement: Supplementary file 1 [file ijms-26-05536-s001.zip › ijms-3561704-supplementary.pdf]

Table S1 List of primers used in this study

Primer[a]	Sequence (5' to 3')[b]
ORF7a-F	GC*GAATTC*AAAATTATTCTTTTCTTGG
ORF7a-R	GC*CTCGAG*TCATTCTGTCTTTCTTTTG
ORF7a-20F	GC*GAATTC*TACCAAGAGTGTGTTAG
ORF7a-55R	GC*CTCGAG*TTATGCAAATTTGTTATCAG
ORF7a-56F	GC*GAATTC*CTGACTTGCTTTAGCAC
ORF7a-95R	GC*CTCGAG*TTATTCTTGAACTTCCTCT
Q-Rpl32-F	ACAAAGCACATGCTGCCCAGTG
Q-Rpl32-R	TTCCACGATGGCTTTGCGGTTC
Q-ISG56-F	CCTCCTTGGGTTCGTCTACA
Q-ISG56-R	GGCTGATATCTGGGTGCCTA
Q-JAK1-F	GAGACAGGTCTCCCACAAACAC
Q-JAK1-R	GTGGTAAGGACATCGCTTTTCCG
Q-JAK2-F	CCAGATGGAAACTGTTCGCTCAG
Q-JAK2-R	GAGGTTGGTACATCAGAAACACC
Q-TYK2-F	GGTTGACCAGAAGGAGATCACC
Q-TYK2-R	TCCTCGTCATCCATCTTGCCCT

[a] F: forward primer, R: reverse primer. “Q” before a primer name indicates that the primer is used for RT-qPCR analysis.

[b] Italicized letters indicate restriction enzyme cleavage sites for EcoRI or XhoI for directional cloning.
